# Identification and Analysis of the Active Phytochemicals from the Anti-Cancer Botanical Extract Bezielle

**DOI:** 10.1371/journal.pone.0030107

**Published:** 2012-01-17

**Authors:** Vivian Chen, Richard E. Staub, Scott Baggett, Ramesh Chimmani, Mary Tagliaferri, Isaac Cohen, Emma Shtivelman

**Affiliations:** BioNovo, Inc., Emeryville, California, United States of America; University Health Network, Canada

## Abstract

Bezielle is a botanical extract that has selective anti-tumor activity, and has shown a promising efficacy in the early phases of clinical testing. Bezielle inhibits mitochondrial respiration and induces reactive oxygen species (ROS) in mitochondria of tumor cells but not in non-transformed cells. The generation of high ROS in tumor cells leads to heavy DNA damage and hyper-activation of PARP, followed by the inhibition of glycolysis. Bezielle therefore belongs to a group of drugs that target tumor cell mitochondria, but its cytotoxicity involves inhibition of both cellular energy producing pathways. We found that the cytotoxic activity of the Bezielle extract *in vitro* co-purified with a defined fraction containing multiple flavonoids. We have isolated several of these Bezielle flavonoids, and examined their possible roles in the selective anti-tumor cytotoxicity of Bezielle. Our results support the hypothesis that a major Scutellaria flavonoid, scutellarein, possesses many if not all of the biologically relevant properties of the total extract. Like Bezielle, scutellarein induced increasing levels of ROS of mitochondrial origin, progressive DNA damage, protein oxidation, depletion of reduced glutathione and ATP, and suppression of both OXPHOS and glycolysis. Like Bezielle, scutellarein was selectively cytotoxic towards cancer cells. Carthamidin, a flavonone found in Bezielle, also induced DNA damage and oxidative cell death. Two well known plant flavonoids, apigenin and luteolin, had limited and not selective cytotoxicity that did not depend on their pro-oxidant activities. We also provide evidence that the cytotoxicity of scutellarein was increased when other Bezielle flavonoids, not necessarily highly cytotoxic or selective on their own, were present. This indicates that the activity of total Bezielle extract might depend on a combination of several different compounds present within it.

## Introduction

Bezielle (BZL101) is an aqueous extract of the aerial parts of the herb Scutellaria barbata long used for treatment of fevers and cancer in traditional Chinese medicine. Bezielle is selectively cytotoxic to tumor cells while sparing normal and non-transformed cells *in vitro* [1]. Bezielle extract had showed a promising anti-cancer activity in early clinical testing [2], [3], but further clinical development of Bezielle would be advanced by the chemical identification of the compound(s) in Bezielle that are directly responsible for its anti-cancer activity. This strategy is the guiding principle of the anti-cancer research conducted at BioNovo that aims to bring to the practice of Western medicine some of the herbal knowledge accumulated in the Chinese traditional medicine. The goal is to bridge between the botanical-based traditional medicine and compound-based Western medicine which, by necessity, involves identification of the active phytochemicals in the total herbal extracts. In this paper we describe the identification and analysis of the active phytochemical(s) in Bezielle. Activity-guided fractionation of Bezielle led to the identification of a distinct fraction that was selectively cytotoxic *in vitro*. Further chemical analysis of this fraction revealed that it contains a number of related compounds belonging to a family of flavonoids. Flavonoids are phytochemicals found in a wide variety of plants, including those common in normal diet. Flavonoids, in general, are considered to have anti-inflammatory, anti-oxidant and cancer-preventive properties (reviewed among others in [4], [5], [6]).

The accompanying paper in this issue and our results published previously [1] show that Bezielle kills tumor cells via induction of reactive oxygen species (ROS), DNA damage, collapse of redox status and metabolic suppression, that involves inhibition of both mitochondrial respiration and glycolysis, as well as depletion of the mitochondrial reserve capacity. All these activities of Bezielle are abolished in tumor cells with disabled mitochondrial respiration. We have therefore examined the Bezielle-derived flavonoids for the activities characteristic of Bezielle. Specifically, four major Bezielle flavonoids identified via activity-guided isolation were analyzed for (a) selective cytotoxicity to cancer cells; (b) ability to induce progressively increasing levels of ROS; (c) dependence of ROS induction and cytotoxicity on presence of respiring mitochondria; (d) induction of DNA damage; (e) effects on cellular redox status; (f) metabolic suppression.

We report that some flavonoids, far from having anti-oxidant properties, induce ROS and DNA damage preferentially in tumor cells. In particular, scutellarein was selectively cytotoxic to tumor cells but not to non-transformed cells. Scutellarein induced progressively higher levels of ROS and DNA damage in a time dependent manner, similar to total Bezielle. Scutellarein also induced metabolic suppression, inhibiting both OXPHOS and glycolysis. The latter activities of scutellarein as well as its cytotoxicity were enhanced when it was combined with other flavonoids that by themselves are not highly cytotoxic and do not induce DNA damage.

## Materials and Methods

### Activity Guided Isolation of Anti-cancer Compounds from Bezielle

Tea-like extracts of *Scutellaria barbata* for the activity-guided isolation were prepared by adding water to the ground, dried herb (10∶1, volume : mass), then bringing the mixture to a boil. The herbal solution was allowed to simmer for 45–60 minutes at approximately 70°C, then suction filtered (Whatman 1 paper filter) to produce the crude tea. An equal volume of acetone was added to the extract to create a precipitate. The acetone:water solution was suction filtered (Whatman 1 paper filter), then concentrated by rotary evaporation *in vacuo* to remove the acetone and further reduce the aqueous volume by 60–70%. The concentrated tea was filtered again (0.45 µm).

The concentrated extract was subjected to open column chromatography over Diaion HP-20 resin (Supelco, Bellefonte, PA). The sample was loaded onto the column in 20% methanol in water and eluted with 20%, 50%, 75% and 100% methanol (three column volumes for each step). Fractions were tested for cytotoxicity using CCK8 assay, and for DNA damaging activity using Comet assay. Both activities were found to be associated with the 75% and 100% methanol fractions.

**Figure 1:**
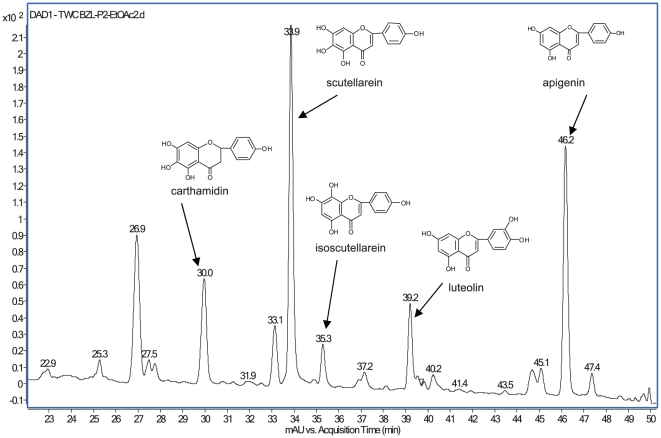
HPLC/MS chromatogram of an active fraction isolated from Bezielle. This fraction was isolated as described in Experimental procedures. Structures of some of the compounds that were further analyzed are shown.

Active fractions from the HP-20 column were combined, concentrated and subjected to open column chromatography over Sephadex LH20 resin (Sigma-Aldrich Chemical Company, Milwaukee, WI). The sample was loaded in 1∶1 methanol—water and eluted in four steps at 50%, 60%, 75%, and 100% methanol in water. Cytotoxicity assay data determined that the greatest activity was in fractions that eluted from the column in 75–100% methanol. A fraction similar in composition and activity was also prepared by partitioning Bezielle with ethyl acetate (Figure 1).

Preparative HPLC was performed on the active fractions that were recovered from the LH20 column or the equivalent ethyl acetate partition of Bezielle. Preparative HPLC employed a linear gradient from 10% to 60% acetonitrile in 0.1% aqueous trifluoroacetic acid over 30 min on a Phenomenex Luna C18(2) column (150×21.1 mm, 5 µm) at a flow rate of 20 mL/min. Several compounds were purified by preparative HPLC and their structures were elucidated. Scutellarein, luteolin and apigenin were identified based on LC/MS and NMR comparison with commercial reference standards. Isoscutellarein and carthamidin were identified based on LC/MS, 1D and 2D NMR and comparison with literature data. Apigenin and luteolin were purchased from Indofine Chemical Company, Inc. (Hillsborough, NJ). Scutellarein was purchased from Apin Chemical Company (Abingdon, Oxfordshire UK). Scutellarein, isoscutellarein and carthamidin were also synthesized at BioNovo. Data for structural elucidation of compounds and synthetic procedures can be found in the Methods S1.

### Reagents and antibodies

All reagents and inhibitors were purchased from Sigma. Fluorescent indicators of ROS, mitochondrial superoxide and nitric oxide, CM-H_2_DCFDA, MitoSox and CM-H_2_DAFDA and were from Molecular Probes/InVitrogen. Antibodies to nitrotyrosine were from Millipore, antibody to PAR was from Becton-Dickinson.

### Cell culture and treatments

All cell lines were obtained from the ATCC, and propagated in RPMI with 10% FCS (MDAMB231), DME with 10% FCS (Hs578T, SKBr3) and DME/F12 with 5%FCS and supplements (MCF10A and 184A1). The medium for MCF10A and 184A1 also contained 50 µg/ml pituitary extract, 10 µg/ml insulin, 0.5 µg/ml hydrocortisone and 0.02 µg/ml EGF (Sigma). All media were pyruvate-free. Cells were treated with BZL101 at a concentration of 250 µg/ml (dry weight per volume) or with water (labeled as “untreated” throughout the paper). Flavonoids were dissolved in DMSO, and control cultures were treated with DMSO alone.

### Measurements of cell viability, mitochondrial potential, ATP and GSH

Cell viability was determined using CCK-8 kit (Dojindo). Mitochondrial transmembrane potential (ΔΨM) in live cells was analyzed using the potential-sensitive cationic dye JC-1 (Molecular Probes) by incubating cells with 2 µM JC-1 for 30 minutes. Cells were washed, and red fluorescence (indicative of healthy mitochondria) as well as green fluorescence (indicative of mitochondria with low ΔΨM) was determined on FACScan. ATP levels were quantified using ATP Bioluminescence assay kit HS II (Roche Applied Science). For determination of the levels of reduced glutathione, cells in 96 well plates were incubated in media containing 8 µM monobromobimane (mBB). At different times after addition of mBB fluorescence was read on a plate reader with filters set at excitation of 360 nM and emission of 460 nM.

### Analysis of oxidative protein modifications

Carbonyl modification of proteins in cells treated with Bezielle and flavonoids was quantified using the FlowSellect Oxidative Stress kit from Millipore. The kit contains 2.4-dinitrophenylhydrazin (DNP), chemical that covalently binds to carbonyl modification on proteins. After derivatization of carbonyl groups, the DNP bound proteins are detected with A FITC-conjugated anti-DPN antibody.

### Western blot analysis

Whole cell lysates were electrophoresed on SDS-PAGE and transferred to nitrocellulose membranes. Membranes were blotted with antibodies at recommended concentrations overnight at 4°C and the bound primary antibodies were detected using peroxidase-conjugated secondary antibodies. Blots were developed using SuperSignal enhanced chemiluminescence kit (Pierce) and imaged on Kodak Imager ISR2000.

### Comet Assay

Alkaline comet assays were performed using the Comet assay kit from Trevigen according to the manufacturer's instructions. After electrophoresis the nuclei were stained with SYBR green and viewed under a fluorescence microscope. Percentages of cells with comets were quantified by an observer blinded to the identity of the slides. Olive tail moment, defined as the product of percentage DNA in the tail and displacement between the position of the mean centers of mass in the heads and tails, was determined for at least 40 cells per sample. Cells were photographed and analyzed with the TriTek CometScore image analysis software.

### Metabolic analyses with Seahorse XF96 Extracellular Flux Analyzer

Cells were plated overnight on XF96 PET 96 well plates at experimentally predetermined numbers (10×10^3^ cells per well for MDAMB231 and 12×10^3^ cells per well for MCF10A). Metabolic fluxes were analyzed on Seahorse XF96 analyzer according to the manufacturer instructions as previously described [7]. The basal ECAR values (in mpH/min), PPR (in pmolH+/min) and OCR values (in pMoles O^2^/min) were measured for 4 cycles. After measuring the basal levels, mitochondrial uncoupler FCCP at 1 µM was automatically injected into the experimental wells for the determination of the maximal mitochondrial respiration, or reserve capacity. Following one cycle of measurements inhibitor of mitochondrial complex III antimycin A at 5 µM was injected into the experimental wells, and another measurement cycle was performed. Each experimental point was an average of 6 to 10 replica wells, and experiments were repeated at least 4 times. Normalization of ECAR, PPR OCR values obtained in XF96 assays was performed using quantification of cellular DNA with the CyQuant assay (Promega) on experimental plates. All the XF96 data are expressed as ECAR or OCR values normalized to DNA content, or as percent of activities relative to these measured in the same experiment in cells kept in full media (after normalization of all data sets).

## Results

### Isolation and purification of active fractions and compounds from Bezielle extract

The activity-guided isolation of active fractions and compounds within the active fractions is described in Materials and Methods. A fraction with high and selective cytotoxic activity was isolated (Figure 1). This fraction, similar to total Bezielle extract, was highly cytotoxic towards breast cancer cell lines MDAMB231 and SKBr3 but did not produce significant cell death in the immortalized non-transformed cell line MCF10A or primary human fibroblasts IMR90 (not shown). As described in the Experimental Procedures, the compounds in the fraction highly enriched for cytotoxic activity were identified as flavonoids. Several of them were purified to homogeneity, identified as described in Methods S1 and analyzed in cellular assays.

### Cytotoxic activity of selected Bezielle flavonoids

**Figure 2:**
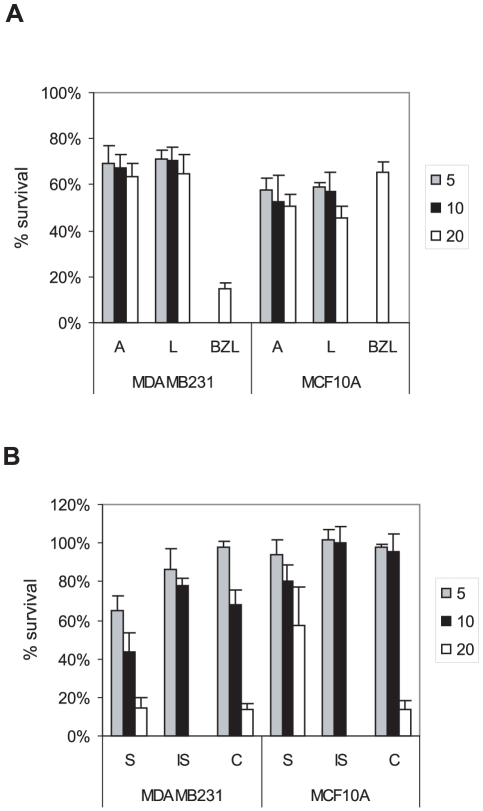
Bezielle flavonoids exhibit differential cytotoxicities towards cancer versus non-transformed cells. **A.** Cytotoxic activity of apigenin (A), luteolin (L) and Bezielle in breast cancer cells MDAMB231 and non-transformed breast epithelial cell line MCF10A. Cells were treated for 24 hours prior to analysis with the concentrations of flavonoids indicated to the right of the charts (5, 10 and 20 µg/ml), or with Bezielle (BZL) at 250 µg/ml. **B.** Cytotoxic activities of scutellarein (S), isoscutellarein (IS) and carthamidin (C), as in **A.** All results are mean ± S.E. (n = 4).

First, the cytotoxicity of several flavonoids isolated from Bezielle was tested with breast cancer lines MDAMB231 and non-transformed mammary cell line MCF10A. Figure 2A shows cytotoxicity of the two closely related flavones apigenin (A) and luteolin (L) that are extensively characterized in the published literature, with most reports describing anti-oxidant properties of these compounds. Clearly, these flavones had a limited cytotoxicity in MDAMB231 cells and a somewhat higher activity towards MCF10A cells, which is just the opposite of the selectivity seen with Bezielle. In addition, the doze response of MCF10A and MDAMB231 cells to A and L was relatively flat over the range of concentrations from 5 to 20 µg/ml (Figure 2A), indicating a lack of dose-response. These results suggest that neither apigenin nor luteolin individually contribute significantly to the selective cytotoxic activity of Bezielle.

Figure 2B shows induction of cell death by the closely related flavones scutellarein (S) and isoscutellarein (IS) as well as the flavonone carthamidin (C). Scutellarein had a limited effect on the viability of MCF10A cells, but was highly cytotoxic towards MDAMB231 (and other cancer cell lines, not shown) in a dose-dependent manner (Figure 2B). Isoscutellarein was significantly less cytotoxic to MDAMB231 cells than scutellarein, and did not kill MCF10A cells, indicative of a limited but selective cytotoxicity. Carthamidin was more active against MDAMB231 cells than MCF10A cells at the two lower concentrations. However, the cell death in cultures treated with 20 µg/ml of carthamidin was very significant and apparent already at 4 to 5 hours after the start of treatment, indicating a severe toxicity of carthamidin at higher concentration.

Two additional breast cancer cell lines, Hs578T and SKBr3 showed the same pattern of sensitivity to the flavonoids as MDAMB231 (Figure S1 and not shown). We have also tested other immortalized non-transformed cell line derived from mammary epithelium, 184A1 and MCF12A, which responded to treatment with flavonoids similarly to MCF10A (not shown).

These results identify scutellarein as the most cytotoxic and, importantly, selectively cytotoxic, Bezielle flavonoid out of the compounds analyzed here. Isoscutellarein showed selectivity towards cancer cells, but was not studied further because of its marginal cytotoxicity. Carthamidin was also selective towards cancer cells at lower concentrations, while apigenin and luteolin were not at all selective.

### Generation of ROS by Bezielle flavonoids

**Figure 3:**
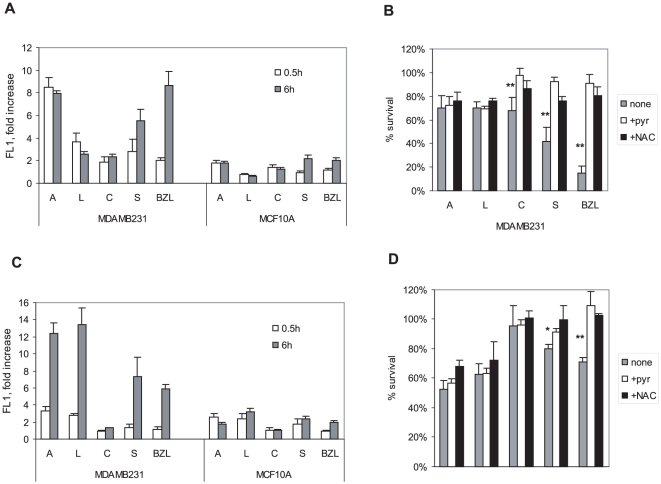
Differential induction of ROS by Bezielle flavonoids. **A.** Induction of peroxide type ROS by the flavonoids in MDAMB231 and MCF10A cells. Cells were treated for 30 minutes or 6 hours with the indicated flavonoids at 10 µg/ml and analyzed for green fluorescence (FL1) on a FACScan after loading with H_2_DCF-DA. Results are shown as fold increase of the FL1 observed in untreated cells analyzed in same experiments (FL1 levels in untreated cells are marked by dotted line). **B.** Induction of mitochondrial superoxide by Bezielle flavonoids. After treatment as in A, cells were loaded with MitoSox Red and analyzed for levels of red fluorescence (FL2). Results are presented in the same way as in A, and are mean ± S.E. of three to five experiments **C.** Survival of MDAMB231 cells treated with flavonoids at 10 µg/ml for 24 hours in the absence or presence of 10 mM pyruvate or NAC. Results are mean ± S.E. (n = 3). Significant differences (** P<0.01, *P<0.05) between cells treated with flavonoids or Bezielle alone or in presence of antioxidants are e are shown. D. Same as in C, with MCF10A cells.

The process of Bezielle induced death in cancer cells is initiated by generation of high levels of ROS. Moreover, the levels of ROS, both peroxide type and mitochondrial superoxide elicited by Bezielle greatly increases over time in tumor cells but not in MCF10A cells (Chen et al., submitted). We therefore examined if individual flavonoids from Bezielle generate ROS and superoxide in cancer cells but not in non-transformed cells, and if induction of ROS increases over time as seen with Bezielle. Levels of peroxide type ROS (detectable with H_2_DCFDA) were examined in treated cells at different times, and two of the time points are shown in Figure 3A. Two distinct features are clear: a) all flavonoids induce much higher levels of peroxide type ROS in MDAMB231 than in MCF10A cells, and b) only scutellarein shows a time-dependent increase in levels of ROS, similar to Bezielle. The induction of peroxide type ROS by apigenin, carthamidin and luteolin did not increase over time, but remained either unchanged, or was somewhat diminished, even though initially apigenin induced highest levels of ROS. The increasing levels of the DNA damaging peroxide type ROS induced by scutellarein in a time dependent manner further supports the possibility that scutellarein is involved in the selective anti-tumor cytotoxicity of Bezielle.

Induction of mitochondrial superoxide examined using the indicator MitoSox Red showed that flavonoids elicit a time-dependent increase in mitochondrial superoxide in MDAMB231 cells (Figure 3B). The increase was very significant (at least 5-fold) for three flavonoids but not for carthamidin, which showed only a 30% increase after 6 hours. Surprisingly, apigenin and luteolin, that were less cytotoxic in MDAMB231 cells, induced the highest levels of mitochondrial superoxide that was further increased in a time-dependent manner (Figure 3B). This was in contrast to the lack of increase in levels of peroxide type ROS over time in cells treated with these flavonoids (Figure 3A). Scutellarein, similar to Bezielle, had a modest effect on mitochondrial superoxide in the beginning of the incubation, but increased the levels of superoxide about seven-fold after 6 hours (Figure 3B).

Induction of ROS by Bezielle is critical for its cytotoxicity, which was abolished in presence of oxidant scavengers such as N-acetylcystein (NAC) and pyruvate [1]. We have examined whether NAC or pyruvate could inhibit cell death induced by individual flavonoids. Both pyruvate and NAC individually inhibited cell death induced by carthamidin and scutellarein in MDAMB231 cells (Figure 3C), suggesting that these compounds induce cell death through oxidative stress, similar to Bezielle. However, neither pyruvate nor NAC could protect MDAMB231 cells from the limited cell death induction by apigenin and luteolin (Figure 3C), indicating that death elicited by these compounds was not a consequence of the oxidative damage.

Similar experiments were conducted with MCF10A cells that are somewhat more sensitive to killing by apigenin and luteolin. As with MDAMB231 cells, there was minimal protection by pyruvate and NAC from death induced by apigenin and luteolin, but a good protection was observed with scutellarein and Bezielle-treated cells (Figure 3D). The experiments with NAC and pyruvate were also performed with a higher concentration of flavonoids (20 µg/ml), and at this concentration cells were also protected from killing by carthamidin and scutellarein, but not apigenin and luteolin (not shown). These results strongly indicate that scutellarein and carthamidin induce cell death via mechanisms involving oxidative stress, whereas apigenin and luteolin, though they elicit cellular ROS, induce limited cell death that is not oxidative in nature.

### Scutellarein induces mostly mitochondrial ROS that are critical for its cytotoxicity

**Figure 4:**
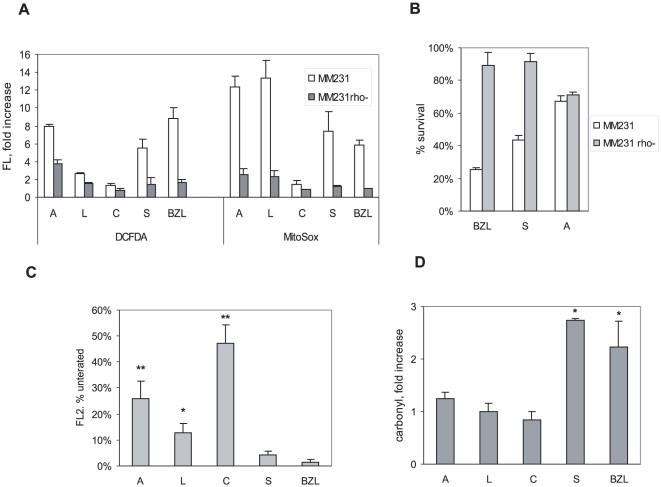
Inhibition of mitochondrial respiration inhibits induction of ROS, DNA damage and cell death by flavonoids. **A.** MDAMB231 and MDAMB231Rho-0 cells were treated with flavonoids for 6 hours, and levels of ROS and mitochondrial superoxide were quantified as in Figure 2. Results are mean ± S.E. (n = 3). **B.** MDAMB231 Rho-0 cells and MDAMB231 cells were treated with Bezielle, scutellarein or apigenin for 24 hours, and percentages of surviving cell quantified. Data are average of three experiments. **C.** Dissipation of mitochondrial transmembrane potential by flavonoids. Cells were treated for 2 hours, loaded with potential-sensitive dye JC-1 and analyzed by flow cytometry. The FL2 fluorescence is reduced in cells with lowered ΔΨM; FL2 of untreated cells was assigned a level of 100%. All treatments resulted in statistically significant dissipation of ΔΨM compared to control cells. Significant differences were also observed between cells treated with scutellarein and other groups, and are indicated (** P<0.01,*P<0.05). **D.** Detection of carbonylated proteins in cells treated with the indicated flavonoids or Bezielle for 4 hours. After treatment cells were fixed, treated with DNP, washed and incubated with a FITC-conjugated antibody to DNP, and analyzed by flow cytometry. All results are mean ± S.E. (n = 3). Significant differences (** P<0.01, *P<0.05) compared to untreated are shown.

Bezielle induces ROS predominantly in mitochondria, because treatment of the Rho-0 variant of MDAMB231 that lacks respiration-competent mitochondria, fails to induce either mitochondrial superoxide or peroxide type ROS. More importantly, MDAMB231 Rho-0 cells are remarkably resistant to the Bezielle induced death (Chen et al., submitted). Therefore, we examined if absence of respiring mitochondria affects ROS induction by individual flavonoids. Figure 4A shows that induction of mitochondrial superoxide by all flavonoids is very strongly attenuated in MDAMB231Rho-0 cells. In particular, superoxide generation by scutellarein and carthamidin was completely inhibited. Similarly, the induction of DCF-DA detectable peroxide type ROS by carthamidin, scutellarein and Bezielle was also almost completely inhibited. This strongly suggest that mitochondria are the primary source of ROS induced by these flavonoids However, apigenin and luteolin still induced significant levels of DCF-DA detectable ROS in Rho-0 cells. This indicates that apigenin and luteolin could target cellular ROS sources other than mitochondria.

We and others have shown that the widely used NOX inhibitor DPI has a strong inhibitory effect on mitochondrial respiration. DPI strongly inhibits Bezielle induced ROS generation and cell death (Chen et al., submitted). We have examined whether DPI is capable of attenuating ROS induction by flavonoids, and observed a pronounced inhibition of ROS, very similar to that observed in Rho-0 cells (Figure S2). Inhibition of respiration in Rho-0 cells and in cells acutely treated with DPI therefore had very similar inhibitory effects on induction of mitochondrial ROS by flavonoids (Figure S2).

We next examined whether inhibition of mitochondrial respiration protects cells from flavonoid-induced death. Scutellarein, similar to Bezielle (Chen et al., submitted), failed to induce cell death in Rho-0 cells (Figure 4B). However, the cell death induction by apigenin (Figure 4B) and luteolin (not shown) was not affected by lack of functional mitochondria.

These results strongly suggest that apigenin and luteolin are not likely to contribute to the oxidative capacity of Bezielle. Even though both flavones induced high levels of mitochondrial superoxide, neither treatment with antioxidants nor disabling mitochondrial respiration affected their limited cytotoxicity. Therefore, the contribution of apigenin and luteolin to the cytotoxicity of Bezielle might not involve ROS. In contrast, cytotoxicity of scutellarein and carthamidin was strongly inhibited either by treatment with ROS scavengers or in Rho-0 cells, similar to Bezielle.

### Flavonoids elicit reduction in the mitochondrial transmembrane potential

We have shown previously that Bezielle induces a strong dissipation of the mitochondrial membrane potential (ΔΨM) in tumor cells [3]. We therefore examined the potential effects of flavonoids on ΔΨM. As seen in Figure 4C, of the four flavonoids tested, scutellarein induced the strongest loss of ΔΨM, while carthamidin was not particularly active, in agreement with the low levels of mitochondrial ROS it induces. Apigenin and luteolin induced dissipation of ΔΨM; however, it was not as dramatic as the loss induced by either scutellarein or Bezielle. We also examined whether the dissipation of mitochondrial transmembrane potential by flavonoids could be attenuated by antioxidants. Figure S3 shows that NAC partially prevented the loss of ΔΨM induced by scutellarein but not by apigenin (and luteolin, not shown). Pyruvate had a similar affect (not shown). Together with the results described above, this strongly suggests that scutellarein (and Bezielle) induce direct oxidative damage in mitochondria of tumor cells. Apigenin and luteolin might elicit cellular responses that involve mitochondria, but might not depend on a direct oxidative damage to mitochondria.

### Induction of protein oxidation

Oxidative stress induces damage of biomolecules other than DNA. We have examined whether Bezielle and its flavonoids induce a common oxidative modification of proteins, carbonylation. Figure 4D shows that Bezielle and scutellarein significant increased the cellular content of carbonylated proteins, and a slight increase was observed with apigenin. Carthamidin and luteolin did not induce oxidative damage to cellular proteins. The induction of protein carbonylation by Bezielle and scutellarein provides an additional confirmation for the hypothesis that scutellarein is the major cytotoxic agent in Bezielle.

### Induction of DNA damage by flavonoids from Bezielle

Bezielle induces oxidative DNA damage that is critical for its ability to selectively kill cancer cells [1]. We have examined which of individual Bezielle flavonoids induce DNA damage. MDAMB231 cells were treated with flavonoids and quantified for two parameters of DNA damage using Comet assay: percent of cells with damaged DNA, and the olive moment (the latter reflects the extent of DNA damage per cell). The flavonoids were tested in Comet assay using concentrations from 5 to 20 µg/ml and incubation times from 30 minutes to 6 hours. Even at the highest concentration tested, 20 µg/ml, apigenin and luteolin did not induce DNA damage detectable in Comet assay (not shown).

**Figure 5:**
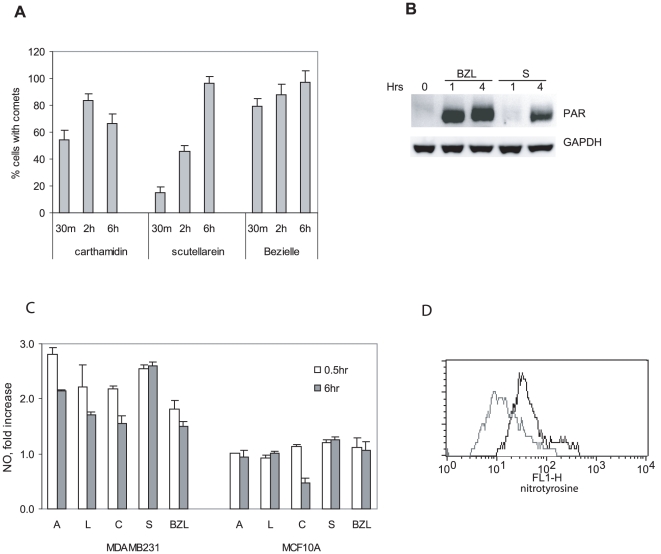
Induction of DNA damage and nitric oxide by flavonoids. **A.** MDAMB231 cells were treated with carthamidin or scutellarein at 10 µg/ml or with Bezielle for different lengths of time and subjected to the alkaline comet assay analysis. Chart shows percentages of cells that formed comets. Results are mean ± S.E. (n = 3). **B.** Detection of PARP activity in MDAMB231 cells treated with Bezielle or scutellarein for 1 and 4 hours. PAR polymers were detected by Western blot analysis. **C.** Induction of nitric oxide by flavonoids. NO was detected using CM-H_2_DAFDA. Cells were treated for 30 minutes or 6 hours. Results are representative of one of the two experiments that produced essentially identical results. **D.** Induction of protein nitrosylation by apigenin. MDAMB231 cells were treated with flavonoids and stained with a specific antibody to nitrotyrosine followed by a secondary fluorescein-conjugated antibody. Cells treated with apigenin for 2 hours showed an increase in fluorescence (gray histogram represents fluorescence of untreated cells; black line corresponds to apigenin-treated).

Scutellarein and carthamidin induced significant DNA damage at concentrations of 5 µg/ml and above. Isoscutellarein also induced DNA damage but only at higher concentrations (not shown). Figure 5A shows that percentage of cells forming comets in presence of scutellarein was gradually increased over time. This correlates with the increasing levels of ROS induced by scutellarein over time (Fig. 3), and is similar to total Bezielle effect. However, Bezielle induced damage in a much higher percentage of cells already within the first 15–30 minutes (Figure 5A). Carthamidin initially induced DNA damage in a higher percentage of cells than scutellarein (Fig. 5A). However, no further increase was observed after the first 2 hours, which correlates with the transient nature of carthamidin induced ROS (Figure 3). Apparently, carthamidin induced massive but relatively transient DNA damage, which indicates that unlike Bezielle- or scutellarein-induced damage, carthamidin-induced DNA damage could be partially repaired over time.

To examine the role of mitochondrial ROS in DNA damage induced by scutellarein and carthamidin, we have performed comet assays in Rho-0 cells as well as in presence of DPI, and inhibitor of mitochondrial respiration and ROS generation. In both cases the extent of DNA damage induced by scutellarein and carthamidin was strongly reduced (Figure S4). Therefore, analysis of DNA damage induced by two flavonoids also supports the hypothesis that scutellarein is the principal selectively cytotoxic flavonoid in the Bezielle extract. Carthamidin induces a somewhat transient DNA damage that could contribute to the cytotoxicity of Bezielle.

We have examined if scutellarein, like Bezielle, induces activation of PARP. Figure 5B shows that scutellarein activates PARP, but this seems to be delayed compared to Bezielle. Bezielle activates PARP within 15 minutes of treatment [1], whereas scutellarein induced appearance of PAR-modified proteins only after 1 hour of treatment. Treatment with apigenin and luteolin predictably had no effect on PARP activity, while carthamidin activated PARP relatively moderately (not shown). We conclude that scutellarein induced DNA damage and activation of PARP are at least in part responsible for the similar activities of Bezielle.

### Induction of nitric oxide by flavonoids

We have addressed the possibility that nitric oxide (NO) could be involved in the induction of DNA damage by Bezielle and flavonoids. Figure 5C shows that all flavonoids induced a modest increase in NO production in MDAMB2231 cells but not in MCF10A. There was no increase in NO levels over time. Having observed an increase in NO levels induced by flavonoids, we have examined the possibility that mitochondrial superoxide and NO could form peroxynitrite, a highly toxic species of ROS capable of damaging DNA and proteins. Staining of treated cells with antibodies to nitrotyrosine revealed that apigenin produced a relatively mild increase in levels of cellular nitrotyrosine (Fig. 5D), but other flavonoids did not increase levels of nitrosylated proteins at any time from 30 minutes to 6 hours of treatments (not shown). We used chemical scavengers of NO and inhibitors of inducible NO synthase in the cytotoxicity assays, and found that they do not protect from cell death induced by Bezielle (not shown). Therefore, induction of NO by flavonoids is unlikely to significantly contribute to the cytotoxicity of Bezielle.

### Glutathione oxidation in cells treated with Bezielle

Because treatment with Bezielle induces a collapse of redox status in tumor cells that manifests itself, among other changes, in depletion of reduced glutathione (GSH), we examined levels of GSH in cells treated with Bezielle flavonoids. The results of these analyses conducted with MDAMB231 cells and MCF10A cells are shown in Figure S5. Luteolin had no significant effect on GSH levels in either of the two cell lines. Apigenin induced a small decrease in GSH in MDAMB231 cells, but had a much more pronounced negative effect on GSH in MCF10A cells. Scutellarein and carthamidin did not influence levels significantly in MCF10A cells, in agreement with their lack of significant cytotoxicity towards these cells. However, scutellarein induced a sustained decrease in GSH levels in MDAMB231 (Figure S5C). Carthamidin reduced GSH levels in a transient manner (Figure S5C), reminiscent of the transient nature of carthamidin induced ROS and DNA damage (Figs. 3 and 5A). We conclude from these data that scutellarein has the most profound effect on ROS levels, DNA damage and redox status of MDAMB231 cells, similar to the total Bezielle extract and indicative of the likely role that this flavonoid plays in the Bezielle cytotoxicity.

### The metabolic effects of flavonoids

**Figure 6:**
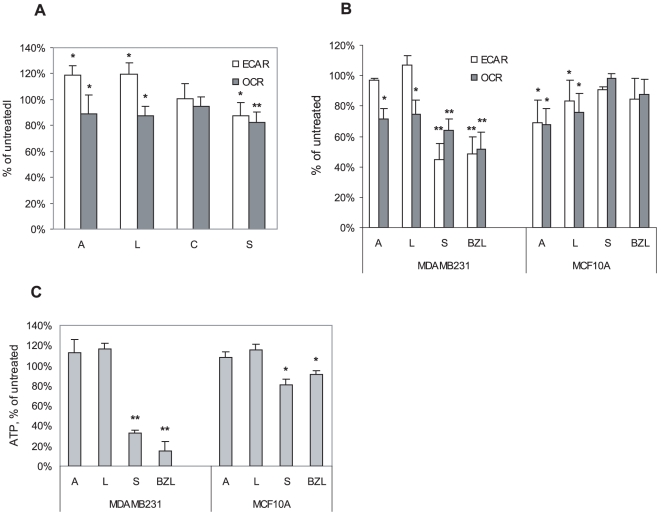
Effect of flavonoids on energy producing metabolic pathways. **A.** Results of metabolic flux analysis of glycolysis (ECAR) and mitochondrial respiration (OCR) in MDAMB231 treated with flavonoids at 10 µg/ml or Bezielle at 300 µg/ml for 4 hours. Results are mean ± S.E. (n = 4). **B.** ECAR and OCR in MDAMB231 and MCF10A cells treated with 20 µg/ml of flavonoids for 4 hours. Results are mean ± S.E. (n = 4). **C.** ATP levels in cells treated as in B. Results are mean ± S.E. (n = 3). Significant differences (** P<0.01, *P<0.05) between treated and untreated groups are shown.

The metabolic effects of Bezielle flavonoids were studied using the Seahorse XF96 metabolic flux analyzer that measures the glycolytic activity (ECAR, a measure of lactate production) and mitochondrial oxygen consumption (OCR) in a real time non-invasive mode. Bezielle induces inhibition of both OCR and ECAR (Chen et al., submitted), first most likely because of oxidative damage to mitochondria, and the latter due to DNA damage, hyper activation of PARP-1 and depletion of cytosolic NAD and ATP. Figure 6A shows that flavonoids had different effects on energy pathways in MDAMB231 cells. At concentrations of 10 µg/ml, apigenin and luteolin showed a statistically significant enhancement of glycolysis and a weak but significant suppression of OCR. The effects of carthamidin were not significant, while scutellarein, similar to Bezielle, suppressed both energy producing pathways. However, the suppression of ECAR and OCR by scutellarein was not as strong as by Bezielle (Figure 6A). We have also examined the effects of flavonoids at 10 µg/ml, on energy producing pathways in MCF10A, and did not observe statistically significant changes (not shown).

We then treated cells with a higher concentration of flavonoids, mainly because cells for the metabolic flux assays are plated at a very high density which in general reduces the inhibitory effects produced by cytotoxic drugs. The negative effects of apigenin and luteolin on OCR (oxygen consumption rate) were somewhat more pronounced, and ECAR (extracellular acidification rate) was largely unaffected by these flavonoids in MDAMB231 cells (Figure 6B). However, the effects of scutellarein were much stronger at the higher concentration consistent with the dose-dependent cell death induced by scutellarein (Figure 2B). Both ECAR and OCR were strongly inhibited by 20 µg/ml scutellarein (Figure 6B). Interestingly, at this concentration scutellarein induced DNA damage in over 80% of cells within the first 15 minutes, similar to total Bezielle (data not shown). Higher concentrations of apigenin and luteolin, but not scutellarein caused a moderate metabolic suppression in MCF10A cells (Figure 6B).

Scutellarein was the only flavonoid tested that, in addition to inhibiting mitochondrial respiration, had a very strong inhibitory effect on ECAR selectively in tumor cells. The inhibition of glycolysis by scutellarein was also seen as reduction in PPR (proton production rate), an independent measure of glycolytic flux (Figure S6A and B). In addition, as shown in Figure S6C, scutellarein, similar to Bezielle, depletes the mitochondrial reserve capacity measured after injection of FCCP that uncouples respiration from ATP synthesis. We conclude that in respect to its metabolic effects, scutellarein shows an activity that is very similar to that of the total Bezielle.

Inhibition of energy producing pathways in cells obviously could lead to ATP depletion. We examined ATP levels in cells treated with flavonoids for 4 hours. Figure 6C shows a very significant reduction of ATP in MDAMB231 cells treated with scutellarein, but not with apigenin and luteolin. The strong depletion of ATP in cells treated with scutellarein and Bezielle was most likely due not only to the inhibition of ATP production, but also to consumption of ATP by the hyper-active PARP-1. Apigenin and luteolin actually induce a transient increase in the ATP levels in MDAMB231 cells, in agreement with the observed increase in glycolytic activity (Figure 6A). Our unpublished results show that induction of death by apigenin and luteolin proceeds along apoptotic pathway that could involve a transient rise in ATP [8].

Even though the effects of scutellarein and Bezielle on metabolic energy producing pathways in MCF10A cells were mild, the ATP levels in these cells were reduced, most likely to support the energy demands of the DNA damage repair pathway (MCF10A show a transient DNA damage after treatment with Bezielle ([1] and Chen et al., submitted).

### Activity of scutellarein is enhanced in presence of other flavonoids

**Figure 7:**
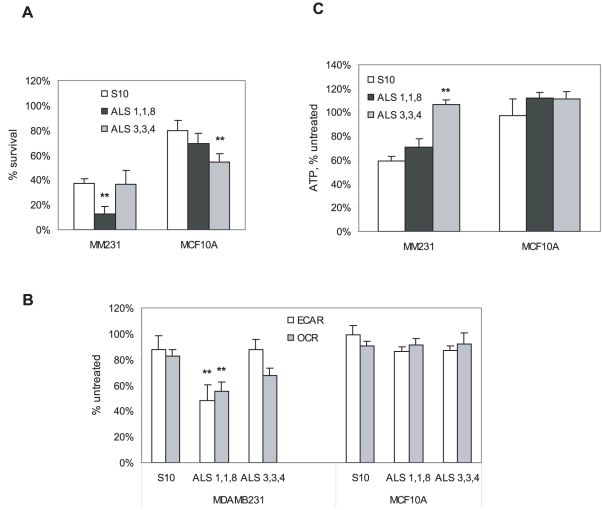
Selective cytotoxicity of scutellarein and its metabolic effects are increased when it is combined with other flavonoids. **A.** Survival of cells treated with the scutellarein alone and combinations of flavonoids at the indicated ratios, all at a final concentration of 10 µg/ml. Mean ± S.E. (n = 3). **B.** Inhibition of OCR and ECAR by scutellarein and mixtures of flavonoids selectively in tumor cells. Cells were treated for 4 hours with scutellarein alone, or with the mixtures of scutellarein with apigenin and luteolin. Mean ± S.E. (n = 4). **C.** ATP levels in cells treated as in B. Results are mean ± S.E. (n = 3). Significant differences (** P<0.01, *P<0.05) between groups treated with mixtures of flavonoids versus scutellarein alone are shown.

Even though scutellarein appears to be the compound responsible for the cancer selective cytotoxicity of Bezielle, the quantitative aspects of this possibility remain uncertain. In addition, we have observed during activity guided isolation that the partially purified fraction depicted in Figure 1 always had a very high activity, while further subfractionation of it into pure compounds did not result in the enrichment of the cytotoxic activity. This was indicative of the existence of additive or even synergistic effects between two or more flavonoids [9]. We have therefore attempted to “reconstitute” the activity of total Bezielle by combining several flavonoids. A number of combinations of flavonoids were tested for cytotoxic activity and compared to scutellarein alone at the same final concentrations. Addition of carthamidin to scutellarein did not influence the cytotoxicity of the latter in either direction, perhaps because both flavonoids induce DNA damage. After testing a number of different combinations we found that a mixture of apigenin, luteolin and scutellarein at 1∶1∶8 ratio (ALS1,1,8) was highly active. Figure 7 shows some of the more detailed analysis of this combination, along with a mixture of the same three flavonoids at the ratio of 3∶3∶4 (ALS3,3,4). ALS118 is significantly more cytotoxic towards MDAMB231 cells than scutellarein alone at 10 µg/ml. Mixture ALS3,3,4 was less cytotoxic to MDAMB231 cells than ALS1,1,8 but killed more MCF10A cells than scutellarein alone (Figure 7A). This indicates that a combination of several flavonoids could be more active than a single flavonoid in terms of selective cytotoxicity, and that the relative abundance of flavonoids in Bezielle is important in determining the selectivity of the extract. In particular, increasing the relative amounts of apigenin and luteolin in the combinations with scutellarein leads to loss of tumor selectivity.

We have addressed the possible basis of the higher selective activity of mixture ALS1,1,8 than of scutellarein alone. We found that ALS1,1,8 mixture was significantly more active in the inhibition of both glycolysis (ECAR) and respiration (OCR) in MDAMB31 cells than scutellarein at the same final concentration (Figure 7B). Importantly, combinations of flavonoids did not induce significant changes in metabolic fluxes of MCF10A cells, indicating that even though mixture ALS3,3,4 induced more cell death in MCF10A, this was not due to metabolic inhibition. This was also reflected in the lack of significant changes in ATP levels in MCF10A cells treated with mixtures of flavonoids, whereas a significant reduction in ATP levels was induced by scutellarein and ALS1,1,8 in MDAMB231 cells (Figure 7C). The mixture ALS3,3,4 did not induce significant loss of ATP in MDAMB231 cells, perhaps indicating that its cytotoxicity involves apoptosis similar to apigenin and luteolin. In addition, ALS 3,3,4 no longer had the scutellarein's selectivity towards tumor cells (Figure 7A).

In conclusion, analysis of the effects of the combination of just three Bezielle flavonoids strongly supports the hypothesis that the selective tumor cell cytotoxicity of Bezielle depends on the multiple flavonoids and their relative amounts in the extract.

## Discussion

The aim of this study was to identify the active phytochemical(s) responsible for the selective cytotoxic activity of Bezielle. We found that the cytotoxic activity of Bezielle *in vitro* co-purifies with a distinct chemically defined fraction that contains a number of polyphenolic phytochemicals known as flavonoids. We set out to analyze Scutellaria flavonoids in a number of assays designed to ascertain whether one or more of the compounds have the following activities determined for Bezielle: (a), induction of ROS, particularly of mitochondrial superoxide; (b) increase in ROS levels over time; (c) induction of oxidative stress induced selective tumor cell death; (d), dependence of cell death induction on presence of respiring mitochondria; (e), induction of DNA damage and hyper-activation of PARP; (f), disruption of cellular redox status; (g), inhibition of both cellular energy-producing pathways.

We identified one flavonoid, scutellarein, which possesses most if not all activities of Bezielle as related to the selective cytotoxicity of the latter. (Table 1). From the analysis of Bezielle we have established that all the processes triggered by it in tumor cells could be traced to the induction of high levels of ROS of mitochondrial origin. Nevertheless, we found that the flavonoids examined here induce various levels of mitochondrial superoxide, but that alone does not confer a selective cytotoxicity to these compounds (Table 1). The distinguishing feature of scutellarein is that, like Bezielle, it induces increasing amounts of both mitochondrial superoxide and peroxide. Probably no less important is the ability of scutellarein to induce increasing levels of DNA damage. Carthamidin at the same concentration also induced DNA damage, but it was transient in nature indicating that it could be repaired. This could explain, at least partially, why carthamidin, at the same concentration as scutellarein, fails to profoundly affect the energy producing pathways in tumor cells.

**Table 1:** Summary of the effects that individual flavonoids produce in tumor cells.

	Apigenin	Luteolin	Carthamidin	Scutellarein	Bezielle
Induction of peroxide ROS	+	+	+	+	+
Induction of mitochondrial superoxide	+	+	−	+	+
Induction of DNA damage	−	−	+	+	+
Induction of protein carbonylation	+/−	−	−	+	+
Depletion of GSH	−	−	+/−	+	+
Inhibition of respiration	+/−	+/−	−	+	+
Inhibition of glycolysis	−	−	−	+	+
Depletion of ATP	−	−	+/−	+	+
Selective cytotoxicity towards tumor cells	−	−	+/−	+	+

Most of the abundant literature published on the activities of apigenin and luteolin describes their antioxidant properties (reviewed in [10], [11], [12]), but a number of publications (to cite just a few of them, [13], [14], [15]) documents their pro-apoptotic activity *in vitro*, including towards normal cells [16]. The reason for these contradictory findings could be that the concentrations used in different publications vary greatly [17]. At concentrations of 5 to 20 µg/ml used in this study, apigenin and luteolin are pro-apoptotic, but show no dose response in their activity (Figure 2A). In addition, both apigenin and luteolin generate high levels of peroxide within minutes after treatment but without further increase, while mitochondrial superoxide showed a time-dependent increase (Figure 3). This could indicate that the mitochondrial superoxide induction by these flavones is secondary to the generation of peroxide type ROS from a different cellular source. It is also possible that some of these observations could be misleading due to the known shortcomings of the available redox-sensitive probes such as used here (reviewed in [18]). However, neither elimination of respiration in Rho-0 cells (Figure 4A), nor acute treatment with DPI, an inhibitor of complex I [19], [20] completely prevents induction of ROS by apigenin and luteolin (Figure 4A and Figure S2). Moreover, the limited death induced by apigenin and luteolin, in either tumor or control cells was not preventable by pre-treatment with either NAC or pyruvate, whereas carthamidin, scutellarein and Bezielle induced death was completely abolished (Figure 3C and 3D). Pyruvate could act as a mitochondrial fuel, and as such could have a dual effect in Bezielle treated cells, but NAC is an antioxidant. All these results strongly suggest that even though mitochondria are apparently targeted by apigenin and luteolin, the limited cytotoxicity of these flavonoids does not rely on their pro-oxidant properties.

We have observed a puzzling discrepancy between the high levels of mitochondrial superoxide induced by apigenin and luteolin (Figure 3B) and lack of DNA damaging or high cytotoxic activity, whereas scutellarein induced lower levels of superoxide that nevertheless lead to damage of nuclear DNA. The fact that scutellarein induced higher levels of peroxide type ROS over time (Figure 3A) could be relevant to its ability to induce increasing DNA damage. It is tempting to speculate that scutellarein might target mitochondrial complex III that, unlike complexes I and II, can produce superoxide on both sides of the mitochondrial inner membrane [21]. Complex III derived superoxide could be released directly into cytoplasm, where it is converted to DNA damaging peroxide [21]. Apigenin and luteolin might target complex I, with superoxide released into mitochondrial matrix and subsequently converted to peroxide that is unable to reach the cell nucleus. This hypothesis is being tested experimentally.

We propose that the high selective cytotoxicity of scutellarein relies on the ability of the ROS that it elicits to disrupt the cellular redox balance, to inhibit OXPHOS and induce DNA damage which leads to the inhibition of glycolysis through hyper activation of PARP. All these activities were observed with the total Bezielle extract (Chen et al., submitted). Carthamidin, while capable of inducing DNA damage, does not affect glycolysis, at least when used at the same concentrations as scutellarein. Apigenin and luteolin induce limited apoptotic death that most likely proceeds through the mitochondrial pathway. Therefore, scutellarein was the only flavonoid of the few analyzed here that induced all the cellular responses associated with Bezielle. The most relevant of these seem to be the ability to progressively increase cellular ROS and induce unrepairable DNA damage with grave consequences for cell metabolic activities.

Nevertheless, we have found that addition of small amounts of apigenin and luteolin to scutellarein results in a higher cytotoxicity in tumor cells than scutellarein alone at the same final concentration. This finding is unexpected for at least two reasons: first, neither apigenin nor luteolin are highly cytotoxic on their own to cancer cell; second, unlike scutellarein, they do not induce either DNA damage or significant inhibition of energy production. Our unpublished results and many published reports show that these flavones induce apoptotic cell death (for example, [15], [16]). We observed annexin V binding, DNA fragmentation (not shown) and transient increase in ATP levels (6C) in tumor cells treated with apigenin or luteolin, all characteristic for the apoptotic cell death. Treatment with scutellarein did not induce hallmarks of apoptotic death (not shown), but was accompanied by the redox and energy collapse characteristic for necrotic death. We show that combining small amounts of apigenin and luteolin with scutellarein increases suppression of OXPHOS (7B). We suggest that this could be relevant to the additive effects of these flavones on induction of cell death.

These findings explain the difficulties we experienced trying to isolate a single phytochemical from Bezielle that had all the relevant activities of the total extract, with the same potency based on its abundance in the extract. Scutellarein has all the relevant activities of Bezielle, but the concentration of scutellarein in total extract is much lower than concentrations used in this study. We show that a combination of just three flavonoids from Bezielle had a greater activity than any individual flavonoid indicating that interactions between the flavonoids resulted in higher cytotoxicity. Our study is not the first to demonstrate positive interactions between flavonoids in respect to cytotoxicity towards cancer cells. Parajuli et al. [9] showed that a combination of four flavonoids from Scutellaria species has a significant cytotoxicity towards cancer cells whereas none of the compounds individually was significantly cytotoxic. Positive interactions between different flavonoids have also been reported in regard to their chemopreventive activities [5], [22], [23].

The active fraction from Bezielle portrayed in Figure 1 is comprised of at least nine identifiable flavonoids, of which we have analyzed here the most prominent ones. The other compounds in the flavonoid enriched fraction are being isolated for further analysis. Finally, and most importantly, *Scutellaria barbata* extracts are known to contain additional cytotoxic compounds such as acteoside/verbascoside [24], as well as other flavonoids, that by virtue of their chemical composition and structure are not constituents of the fraction purified through activity-guided isolation (Figure 1). These compounds might have a somewhat limited cytotoxicity of their own, but in the context of the whole extract in combination with scutellarein, they might have an additive or even synergistic effect. In addition, Bezielle is known to contain much higher concentrations of glucuronidated and glycosylated forms of flavonoids such as scutellarin [25], and others, that have low intrinsic cytotoxicities *in vitro* (not shown). An important point, though not relevant to the *in vitro* studies, is that glucuronidated flavonoids in Bezielle are reduced *in vivo* to aglycons by gut microflora [26], which unmasks their latent cytotoxic activity. Therefore, scutellarin and other conjugated flavonoids in Bezielle could serve as a much larger reservoir for active aglycons *in vivo*.

In summary, this study identified scutellarein as a flavonoid that has most if not all activities relevant to the selective cytotoxicity of Bezielle. We also show that addition to scutellarein of small amounts of other flavonoids that have limited intrinsic cytotoxicity increases the activity of scutellarein. We suggest that these interactions between flavonoids could be directly relevant to the high selective cytotoxicity of Bezielle. The latter might depend on its composition of phytochemicals that produce a variety of cellular responses of which some contribute to selective cytotoxicity if Bezielle.

## Supporting Information

Figure S1 **Breast cancer cell line Hs578T shows a flavonoid sensitivity pattern similar to MDAMB231.** Survival of Hs578T cells treated with 10 µg/ml of the indicated flavonoids for 24 hours. Results are mean +/− s.e. of three experiments.
(PDF)

Figure S2 **DPI inhibits production of ROS.** Generation of ROS in MDAMB231 cells treated with the indicated compounds or Bezielle for 6 hours in absence or presence of DPI (0.75 µM). Cells were analyzed for the peroxide type ROS with DCF-DA and for mitochondrial superoxide with MitoSox.
(PDF)

Figure S3 **Antioxidant attenuates dissipation of DYM induced by scutellarein but not apigenin.** Flow cytometric analysis of MDMA231 cells, untreated (UT) or treated with 10 µg/ml apigenin (A) or scutellarein (S) for 2 hours in absence or presence of 10 mM N-acetylcystein , and then loaded with JC-1, a mitochondrial membrane potential sensitive dye. Percents marked to the right of the plots indicate percentages of cells with high FL2 (i.e., with “normal” values of red fluorescence for JC1 seen in untreated cells normal ΔΨM).
(PDF)

Figure S4 **DPI attenuates DNA damage induced by flavonoids.** Olive moment in MDAMB231 cells treated for 6 hours with either 10 µg/ml of carthamidin or scutellarein, or with 250 µg/ml of Bezielle in presence or absence of 0.75 mM DPI.
(PDF)

Figure S5 **Scutellarein induces depletion of GSH in MDAMB231 but not in MCF10A cells.** The four panels show results of GSH quantification in two cell lines treated with 10 µg/ml flavonoids for the indicated times. Results are expressed as percent GSH of untreated control cells, and are mean ± S.E. (n = 3).
(PDF)

Figure S6 **Effect of scutellarein on metabolic fluxes.** MDAMB231 cells were untreated (UT) or treated with 20 µg/ml scutellarein for 4 hours (S), and analyzed in the Seahorse instrument for the glycolitic activity measured as ECAR (A) and PPR (B) and for mitochondrial respiration, or OCR (C). Injection of mitochondrial uncoupler FCCP was used to measure mitochondrial reserve defined as maximal respiration capacity. Increases in glycolitic rate after injection of FCCP are due to the feedback upregulation of glycolysis by loss of ATP as a result of the block in mitochondrial ATP synthesis induced by FCCP. Injection of Antimycin A was used to confirm that the observed consumption of oxygen is of mitochondrial origin.
(PDF)

Methods S1 **Supplemental experimental procedure.**
(PDF)
