# Supplementary material for: Identification and Analysis of the Active Phytochemicals from the Anti-Cancer Botanical Extract Bezielle
Source: PLoS One. 2012 Jan 17;7(1):e30107. doi: 10.1371/journal.pone.0030107 (PMC3260194; doi:10.1371/journal.pone.0030107)
Supplement: Figure S1 — Breast cancer cell line Hs578T shows a flavonoid sensitivity pattern similar to MDAMB231. Survival of Hs578T cells treated with 10 µg/ml of the indicated flavonoids for 24 hours. Results are mean +/− s.e. of three experiments. (PDF) [file pone.0030107.s001.pdf]

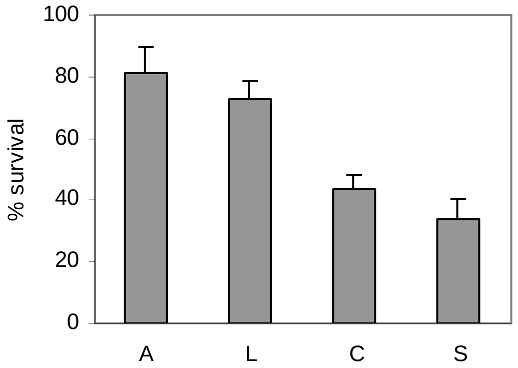

Figure S1. Breast cancer cell line Hs578T shows a flavonoid sensitivity pattern similar to MDAMB231.
Survival of Hs578T cells treated with 10 μg/ml of the indicated flavonoids for 24 hours. Results are mean +/- s.e. of three experiments.
