# Supplementary material for: Identification and Analysis of the Active Phytochemicals from the Anti-Cancer Botanical Extract Bezielle
Source: PLoS One. 2012 Jan 17;7(1):e30107. doi: 10.1371/journal.pone.0030107 (PMC3260194; doi:10.1371/journal.pone.0030107)
Supplement: Figure S2 — DPI inhibits production of ROS. Generation of ROS in MDAMB231 cells treated with the indicated compounds or Bezielle for 6 hours in absence or presence of DPI (0.75 µM). Cells were analyzed for the peroxide type ROS with DCF-DA and for mitochondrial superoxide with MitoSox. (PDF) [file pone.0030107.s002.pdf]

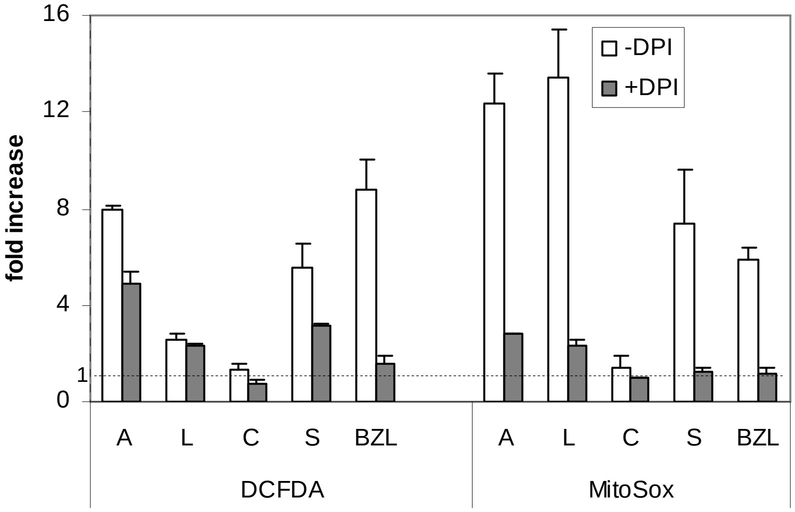

Figure S2. DPI inhibits production of ROS.
Generation of ROS in MDAMB231 cells treated with the indicated compounds or Bezielle for 6 hours in absence or presence of DPI (0.75 μM). Cells were analyzed for the peroxide type ROS with DCF-DA and for mitochondrial superoxide with MitoSox.
