# Supplementary material for: Identification and Analysis of the Active Phytochemicals from the Anti-Cancer Botanical Extract Bezielle
Source: PLoS One. 2012 Jan 17;7(1):e30107. doi: 10.1371/journal.pone.0030107 (PMC3260194; doi:10.1371/journal.pone.0030107)
Supplement: Figure S3 — Antioxidant attenuates dissipation of DYM induced by scutellarein but not apigenin. Flow cytometric analysis of MDMA231 cells, untreated (UT) or treated with 10 µg/ml apigenin (A) or scutellarein (S) for 2 hours in absence or presence of 10 mM N-acetylcystein , and then loaded with JC-1, a mitochondrial membrane potential sensitive dye. Percents marked to the right of the plots indicate percentages of cells with high FL2 (i.e., with “normal” values of red fluorescence for JC1 seen in untreated cells normal ΔΨM). (PDF) [file pone.0030107.s003.pdf]

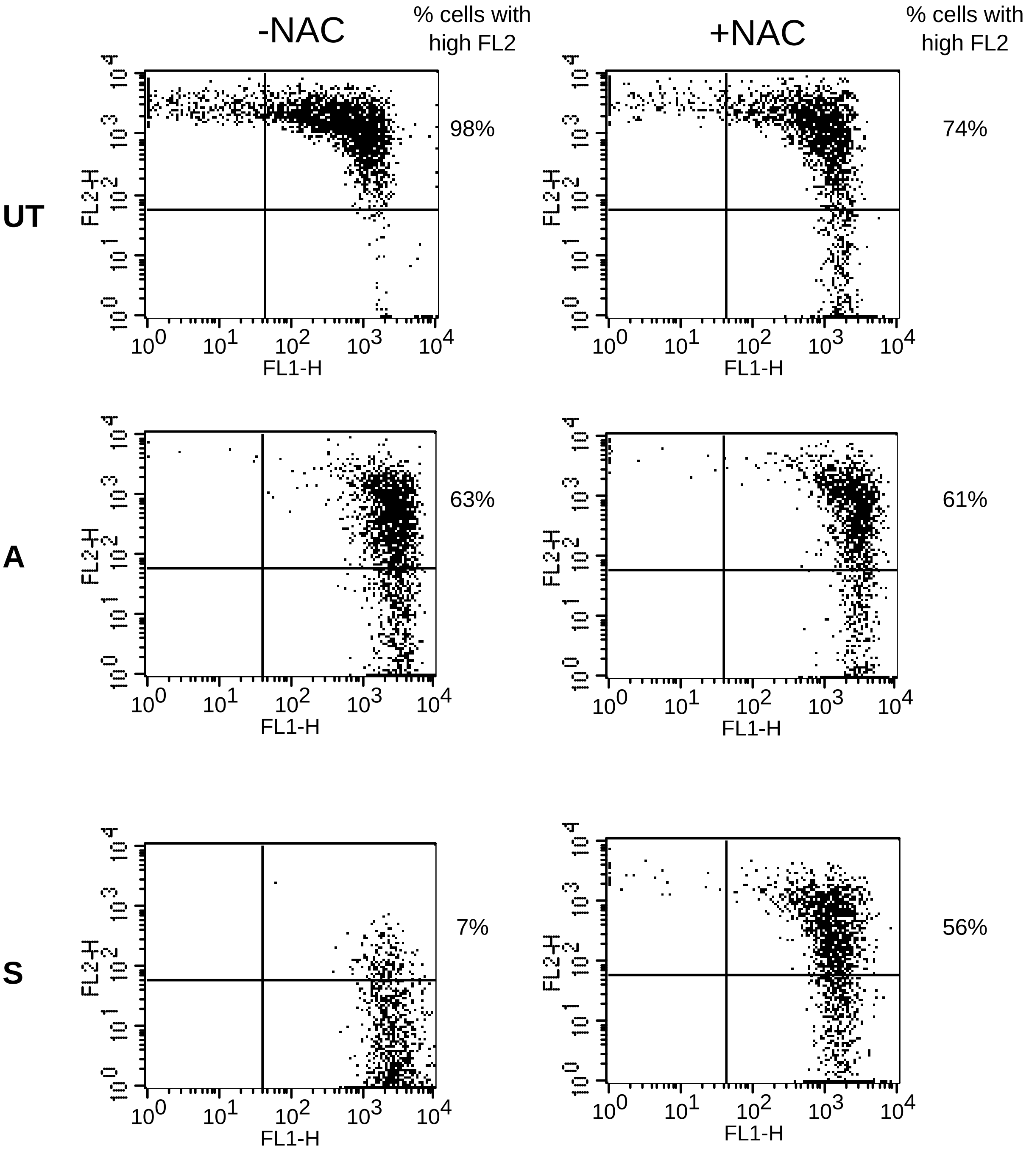

Figure S3. Antioxidant attenuates dissipation of ΔΨM induced by scutellarein but not apigenin. Flow cytometric analysis of MDMA231 cells, untreated (UT) or treated with 10 µg/ml apigenin (A) or scutellarein (S) for 2 hours in absence or presence of 10mM N-acetylcystein , and then loaded with JC-1, a mitochondrial membrane potential sensitive dye. Percents marked to the right of the plots indicate percentages of cells with high FL2 (i.e., with "normal" values of red fluorescence for JC1 seen in untreated cells normal ΔΨM).
