# Supplementary material for: Identification and Analysis of the Active Phytochemicals from the Anti-Cancer Botanical Extract Bezielle
Source: PLoS One. 2012 Jan 17;7(1):e30107. doi: 10.1371/journal.pone.0030107 (PMC3260194; doi:10.1371/journal.pone.0030107)
Supplement: Figure S4 — DPI attenuates DNA damage induced by flavonoids. Olive moment in MDAMB231 cells treated for 6 hours with either 10 µg/ml of carthamidin or scutellarein, or with 250 µg/ml of Bezielle in presence or absence of 0.75 mM DPI. (PDF) [file pone.0030107.s004.pdf]

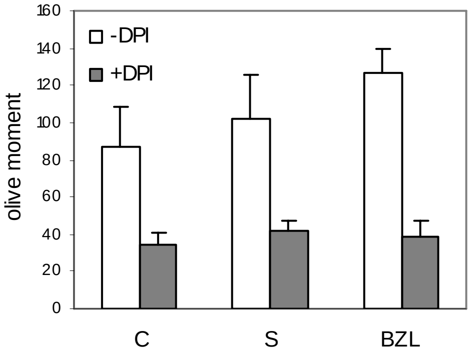

Figure S4. DPI attenuates DNA damage induced by flavonoids.
Olive moment in MDAMB231 cells treated for 6 hours with either 10 μg/ml of carthamidin or scutellarein, or with 250 μg/ml of Bezielle in presence or absence of 0.75 μM DPI.
