# Supplementary material for: Identification and Analysis of the Active Phytochemicals from the Anti-Cancer Botanical Extract Bezielle
Source: PLoS One. 2012 Jan 17;7(1):e30107. doi: 10.1371/journal.pone.0030107 (PMC3260194; doi:10.1371/journal.pone.0030107)
Supplement: Figure S5 — Scutellarein induces depletion of GSH in MDAMB231 but not in MCF10A cells. The four panels show results of GSH quantification in two cell lines treated with 10 µg/ml flavonoids for the indicated times. Results are expressed as percent GSH of untreated control cells, and are mean ± S.E. (n = 3). (PDF) [file pone.0030107.s005.pdf]

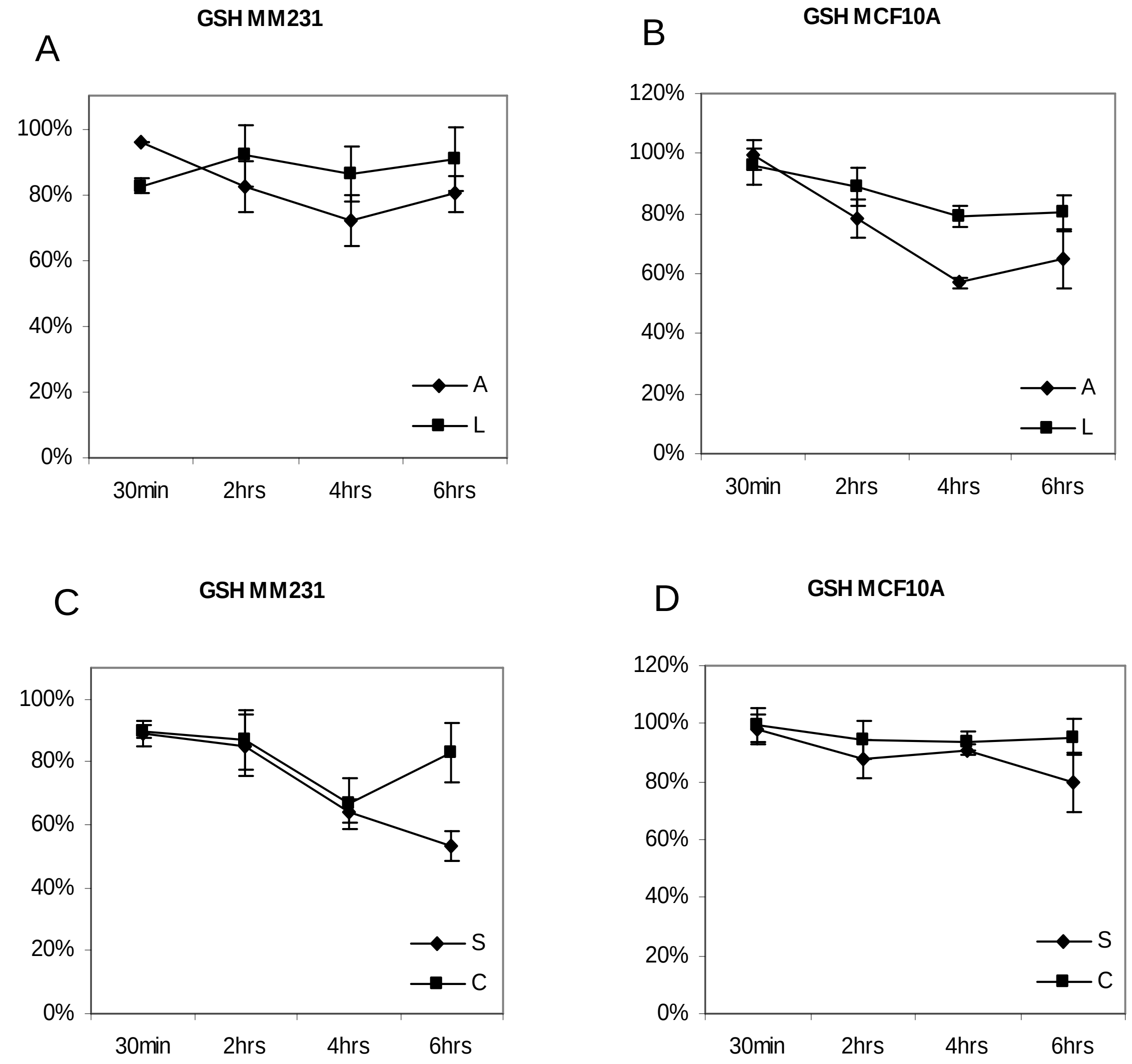

Figure S5. **Scutellarein induces depletion of GSH in MDAMB231 but not in MCF10A cells**. The four panels show results of GSH quantification in two cell lines treated with 10 μg/ml flavonoids for the indicated times. Results are expressed as percent GSH of untreated control cells, and are mean ± S.E. (n=3).
