# Supplementary material for: Identification and Analysis of the Active Phytochemicals from the Anti-Cancer Botanical Extract Bezielle
Source: PLoS One. 2012 Jan 17;7(1):e30107. doi: 10.1371/journal.pone.0030107 (PMC3260194; doi:10.1371/journal.pone.0030107)
Supplement: Figure S6 — Effect of scutellarein on metabolic fluxes. MDAMB231 cells were untreated (UT) or treated with 20 µg/ml scutellarein for 4 hours (S), and analyzed in the Seahorse instrument for the glycolitic activity measured as ECAR (A) and PPR (B) and for mitochondrial respiration, or OCR (C). Injection of mitochondrial uncoupler FCCP was used to measure mitochondrial reserve defined as maximal respiration capacity. Increases in glycolitic rate after injection of FCCP are due to the feedback upregulation of glycolysis by loss of ATP as a result of the block in mitochondrial ATP synthesis induced by FCCP. Injection of Antimycin A was used to confirm that the observed consumption of oxygen is of mitochondrial origin. (PDF) [file pone.0030107.s006.pdf]

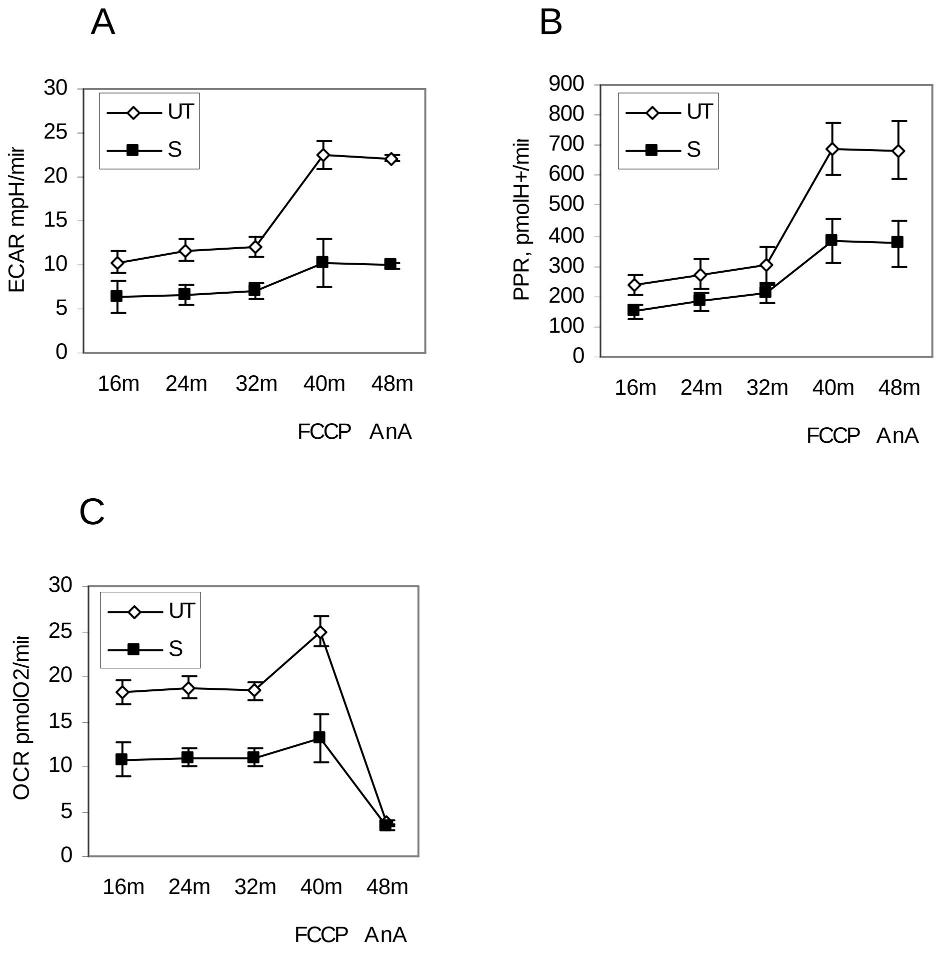

Figure S6. Effect of scutellarein on metabolic fluxes.
MDAMB231 cells were untreated (UT) or treated with 20 μg/ml scutellarein for 4 hours (S), and analyzed in the Seahorse instrument for the glycolitic activity measured as ECAR (A) and PPR (B) and for mitochondrial respiration, or OCR (C). Injection of mitochondrial uncoupler FCCP was used to measure mitochondrial reserve defined as maximal respiration capacity. Increases in glycolitic rate after injection of FCCP are due to the feedback upregulation of glycolysis by loss of ATP as a result of the block in mitochondrial ATP synthesis induced by FCCP. Injection of Antimycin A was used to confirm that the observed consumption of oxygen is of mitochondrial origin.
