# Supplementary material for: Identification and Analysis of the Active Phytochemicals from the Anti-Cancer Botanical Extract Bezielle
Source: PLoS One. 2012 Jan 17;7(1):e30107. doi: 10.1371/journal.pone.0030107 (PMC3260194; doi:10.1371/journal.pone.0030107)
Supplement: Methods S1 — Supplemental experimental procedure. (PDF) [file pone.0030107.s007.pdf]

SUPPLEMENTAL MATERIALS
IDENTIFICATION AND ANALYSIS OF THE ACTIVE PHYTOCHEMICALS FROM THE ANTI-CANCER BOTANICAL EXTRACT BEZIELLE.

Vivian Chen, Richard E. Staub, Scott Baggett, Ramesh Chimmani, Mary Tagliaferri, Isaac Cohen and Emma Shtivelman

SUPPLEMENTAL EXPERIMENTAL PROCEDURES

Spectral data supporting compounds identified from Bezielle: NMR spectra were recorded using a Varian Mercury Plus 400 MHz spectrometer. LC/MS analysis were performed on an Agilent Technologies 1200 Series HPLC system equipped with a diode array detector and a 6210 TOF mass spectrometer operated in the negative mode.

Scutellarein, 5,6,7,4'-tetrahydroxyflavone: CAS# 529-53-3; LC/MS $[M-H]^-$ *m/z* 285.0425; ^{1}H-NMR (pyridine-d_5, 400 MHz) δ 7.95 (2H, *d*, *J* = 8.8, H-2',6'), 7.24 (2H, *d*, *J* = 8.8, H-3',5'), 7.06 (1H, *s*, H-8), 6.93 (1H, *s*, H-3); ^{13}C-NMR (pyridine-d_5, 100 MHz) δ 183.6 (C-4), 164.9 (C-2), 163.0 (C-4'), 155.9 (C-7), 151.6 (C-6), 148.9 (C-5), 131.7 (C-9), 129.3 (C-2',C-6'), 123.2 (C-1'), 117.3 (C-3',C-5'), 105.8 (C-10), 103.8 (C-3), 95.6 (C-8). Data compared with reference standard purchased from Apin Chemical Company and compared to data from literature (1).

Apigenin, 5,7,4'-trihydroxyflavone: CAS# 520-36-5; LC/MS $[M-H]^-$ *m/z* 269.04479. ^{1}H-NMR (DMSO-d_6, 400 MHz) δ 7.93 (2H, *d*, *J* = 8.4, H-2',6'), 6.93 (2H, *d*, *J* = 8.4, H-3',5'), 6.79 (1H, *s*, H-3), 6.49 (1H, *d*, *J* = 2.4, H-8), 6.20 (1H, *d*, *J* = 2.4, H-6); ^{13}C-NMR (DMSO-d_6, 100 MHz) δ 182.2 (C-4), 164.6 (C-7), 164.2 (C-2), 161.9 (C-5), 161.6 (C-4'), 157.8 (C-9), 128.9 (C-2',C-6'), 121.6 (C-1'), 116.4 (C-3',C-5'), 104.1 (C-10), 103.2 (C-3), 99.3 (C-6), 94.4 (C-8). Data compared with reference standard purchased from Indofine Chemical Company.

Luteolin, 5,7,3',4'-tetrahydroxyflavone: CAS# 491-70-3; LC/MS $[M-H]^-$ *m/z* 285.0336; ^{1}H-NMR (DMSO-d_6, 400 MHz) δ 7.41 (1H, *dd*, *J* = 2.4,8.0, H-6'), 7.39 (1H, *d*, *J* = 2.4, H-2'), 6.88 (1H, *d*, J = 8.0 H-5'), 6.67 (1H, *s*, H-3), 6.44 (1H, *d*, J = 2.0 H-8), 6.18 (1H, *d*, J = 2.0 H-6); ^{13}C-NMR (DMSO-d_6, 100 MHz) δ 181.7 (C-4), 164.1 (C-7), 163.9 (C-2), 161.5 (C-5), 157.3 (C-9), 149.7 (C-4'), 145.7 (C-3'), 121.5 (C-1'), 119.0 (C-6'), 116.0 (C-5'), 113.4 (C-2'), 103.7 (C-10), 102.9 (C-3), 98.8 (C-6), 93.8 (C-8). Data compared with reference standard purchased from Indofine Chemical Company and data from literature (2).

Isoscutellarein, 5,7,8,4'-tetrahydroxyflavone: CAS# 41440-05-5; LC/MS $[M-H]^-$ *m/z* 285.04104; ^{1}H-NMR ($CDCl_3$, 400 MHz) δ 7.94 (2H, *d*, *J* = 9.2, H-2',6'), 6.92 (2H, *d*, *J* = 8.8, H-3',5'), 6.55 (1H, *s*, H-3), 6.27 (1H, *s*, H-6); ^{13}C-NMR ($CDCl_3$, 100 MHz) δ 182.8 (C-4), 164.7 (C-2), 161.2 (C-4'), 153.7 (C-7), 153.4 (C-5), 145.7 (C-9), 128.4 (C-2',C-6'), 124.9 (C-8), 122.0 (C-1'), 115.4 (C-3',C-5'), 103.4 (C-10), 101.8 (C-3), 98.2 (C-6). Data compared with data from literature (3).

Carthamidin, 5,6,7,4'-tetrahydroxyflavonone: CAS# 479-54-9; LC/MS $[M-H]^-$ *m/z* 287.0588; ^{1}H-NMR (methanol-d_4, 400 MHz) δ 7.31 (2H, *d*, *J* = 8.0, H-2',6'), 6.81 (2H, *d*, *J* = 8.0, H-3',5'), 5.95 (1H, *s*, H-8), 5.28 (1H, dd, *J* = 2.8, 13.6, H-1), δ 3.08 (1H, dd, *J* = 2.8, 16.8, H-1), δ 2.67 (1H, dd, *J = 4.4*, 16.8, H-1); ^{13}C-NMR (methanol-d_4, 100 MHz) δ 197.1 (C-4), 157.8 (C-4'), 156.0 (C-9), 155.2 (C-7), 149.7 (C-5), 129.1 (C-1'), 127.5 (C-2',C-6'), 126.1 (C-6), 114.8 (C-3',C-5'), 101.8 (C-10), 94.4 (C-8), 79.2 (C-2), 42.8 (C-3). Data compared with data from literature (4)

Synthesis of Scutellarein: 1-(6-hydroxy-2,3,4-trimethoxyphenyl)-3-(4-methoxyphenyl) prop-2-en-1-one (**3**):A mixture of 2,3,5-trimethoxyphenol **1** (5.0 g, 27.0 mmol) and 4-methoxy cinnamyl chloride **2** (5.12 g, 26.0 mmol) was dissolved in BF_3-Et_2O complex (25 mL). The solution was heated to reflux for 30 min. then cooled to room temperature and quenched with an excess of water. After filtration, the product was recrystallized from hexane: EtOAc (3:1), yielding chalcone **3** (6.89 g, 77%) as a deep yellow viscous mass. ^{1}H-NMR (pyridine-D_5) δ 8.17 (1H, d, *J*=15.6 Hz), 8.07 (1H, d, *J*=15.2 Hz), 7.74 (2H, d, *J*=8.4 Hz), 7.04 (2H, d, *J*=8.4 Hz), 6.61 (1H, s), 3.97 (3H, s), 3.88 (3H, s), 3.78 (3H, s), 3.71 (3H, s).

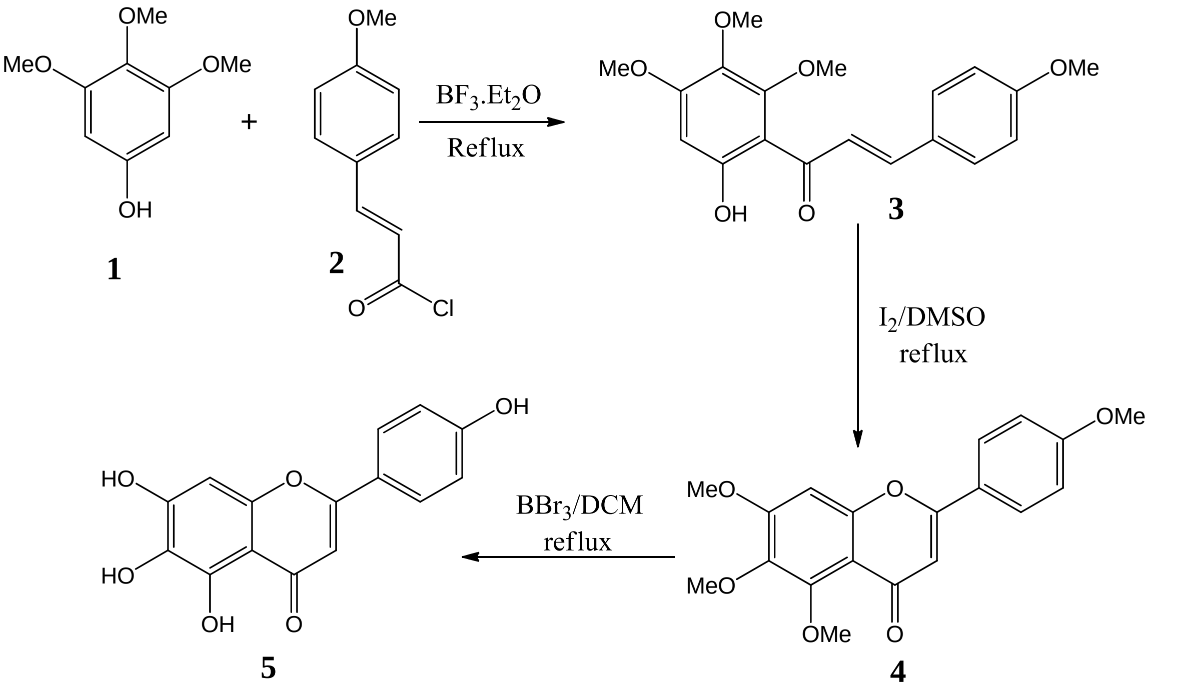

Scutellarein

4′,5,6,7, -Tetramethoxy Flavone (**4**): A mixture of **3** (2.5 g, 7.3 mmol) and iodine (200 mg) in DMSO (10.0 mL) was refluxed for 2 h, then carefully poured onto crushed ice (100g). The precipitate was filtered and washed with 20% Na_2SO_3 then purified by flash column chromatography using hexance-EtOAc=3:1), yielding 1.82 g (73%) of **4** as a pale yellow solid. ^{1}H-NMR (CD_3-OD) δ 7.92 (2H, d, *J*=8.8 Hz), 7.06 (2H, d, *J*=8.8 Hz), 7.05 (1H, s), 6.58 (1H, s) 3.99 (3H, s), 3.91 (3H, s), 3.87 (3H, s), 3.85 (3H, s).

Scutellarein (**5**): To a solution of **4** (1.8 g, 5.2 mmol) in CH_2Cl_2 (40 mL), 1.0 M BBr_3 in CH_2Cl_2 (20.0 mL) was slowly added, then refluxed for 2 h. After cooling to room temperature the reaction mixture was quenched with water and extracted with CH_2Cl_2, then 5% MeOH and EtOAc. Both organic layers were dried on $MgSO_4$ and evaporated under reduced pressure. The resulting pale yellow precipitate was recrystallized from MeOH, yielding 1.12 g pale yellow solid (80%) of Scutellarein **(5)**. ^{1}H-NMR (CD_3-OD) δ 7.82 (2H, d, *J*=9.2 Hz), 6.95 (2H, d, *J*=9.2 Hz), 6.59 (1H, s), 6.55 (1H, s); ^{13}NMR (Pyridine): 183.63, 163.88, 163.00, 155.95, 151.58, 148.92, 131.69, 129.3, 123.20, 117.31, 105.84, 105.82, 103.81, 95.56; MS m/z: 285 (M-H^+).

Synthesis of Isoscutellarein: 5,7,8-trimethoxy-2-(4-methoxyphenyl)-4H-chrome-4-one (**8**): To a stirred solution of 2′-hydroxy-3′,4′6′-Trimethoxy acetophenone (**6**) (750 mg, 3.3 mmol) and 4-methoxy benzaldehyde (**7**) (646 mg, 4.75 mmol) in absolute ethanol (30 mL) was added 30 mL of 50% aqueous KOH. The resulting mixture was stirred at room temperature for 48 h. The reaction mixture was acidified at 0 ^{o}C with 10% aqueous HCl and then extracted with Et_2O (3 x 150 mL). The combined ethereal extracts were washed with brine, dried over anhydrous $MgSO_4$, filtered, and concentrated. The resulting orange yellow solid residue was purified *via* column chromatography on Silica Gel (elution with hexance-EtOAc, 8:2) to give an orange yellow solid 1.01g (20.8 mmol) of **8** (85%).^{1}H-NMR (pyridine-d_5): δ 8.17 (1H, d, *J*=15.6 Hz), 8.07 (1H, d, *J*=15.2 Hz), 7.74 (2H, d, *J*=8.4 Hz), 7.04 (2H, d, *J*=8.4 Hz), 6.61 (1H, s), 3.97 (3H, s), 3.88 (3H, s), 3.78 (3H, s), 3.71 (3H, s).

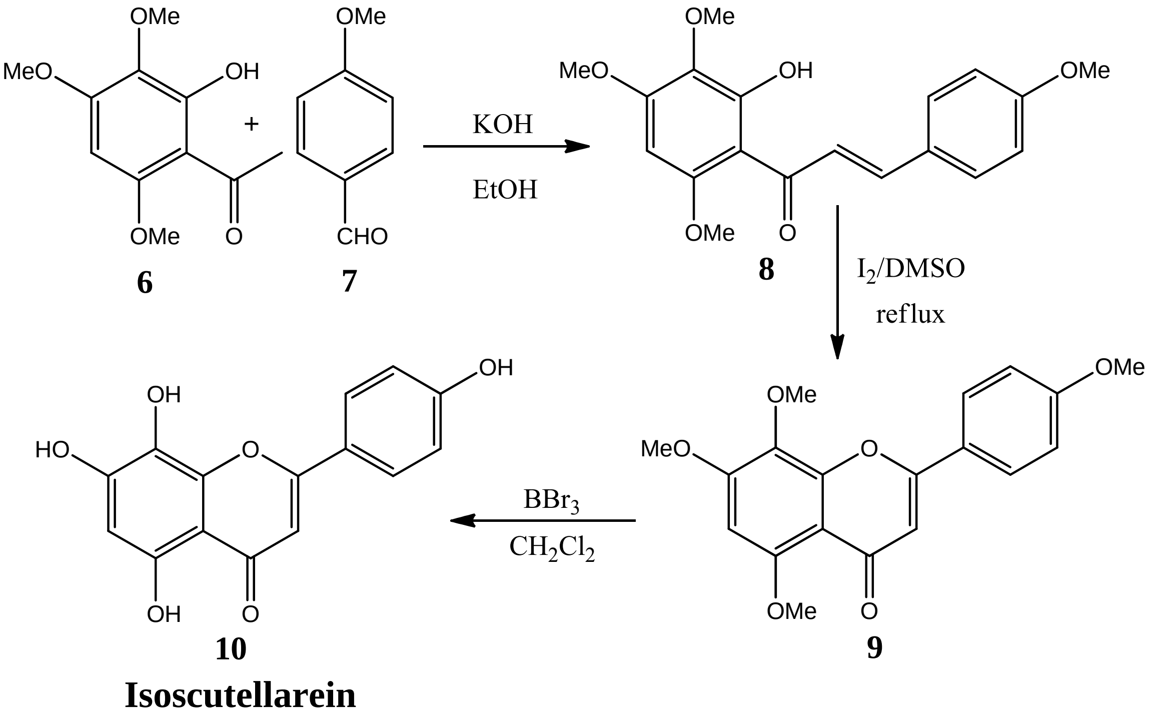

4′,5,7,8 -Tetramethoxy Flavone (**9**): A mixture of **8** (1.0 g, 2.9 mmol) and iodine (200 mg) in DMSO (5.0 mL) was refluxed for 2 h, and then carefully poured onto crushed ice (50 g). The precipitate was filtered and washed with 20% Na_2SO_3 then purified by flash column chromatography on Silica Gel (elution with hexance-EtOAc, 3:1, yielding 0.8 g (80%) of **9** as a pale yellow solid.

Isoscutellarein (**10**): To a solution of **9** (800 mg, 2.3 mmol) in CH_2Cl_2 (40 mL), 1.0 M solution of BBr_3 in CH_2Cl_2 (18.0 mL) was slowly added, then refluxed for 2 h. After cooling to room temperature the reaction mixture was quenched with water and extracted with CH_2Cl_2 then 5% MeOH and EtOAc. Both organic layers were dried on $MgSO_4$ and evaporated under reduced pressure. The resulting pale yellow precipitate was recrystallized from MeOH, yielding 540 mg pale yellow solid (81%) of crude Scutellarein **(10)** which was further purified using HPLC. ^{1}H-NMR (CD_3-OD): δ 7.82 (2H, d, *J*=9.2 Hz), 6.95 (2H, d, *J*=9.2 Hz), 6.59 (1H, s), 6.55 (1H, s); ^{13}NMR (Pyridine): 183.63, 163.88, 163.00, 155.95, 151.58, 148.92, 131.69, 129.3, 123.20, 117.31, 105.84, 105.82, 103.81, 95.56; MS m/z: 285 $[M-H]^-$.

Synthesis of Carthamidin and Isocarthamidin: 1-(6-hydroxy-2,3,4-trimethoxyphenyl)-3-(4-methoxyphenyl) prop-2-en-1-one (1**3**): A mixture of 2,3,5-trimethoxyphenol **11** (5.0 g, 27.0 mmol) and 4-methoxy cinnamyl chloride **12** (5.12 g, 26.0 mmol) was dissolved in BF_3-Et_2O complex (25 mL). The solution was heated to reflux for 30 min. then cooled to room temperature and quenched with an excess of water. After filtration, the product was recrystallized from hexane: EtOAc (3:1) gave chalcone **13** (6.89 g, 77%) as a deep yellow viscous mass. ^{1}H-NMR (pyridine-D_5) δ 8.17 (1H, d, *J*=15.6 Hz), 8.07 (1H, d, *J*=15.2 Hz), 7.74 (2H, d, *J*=8.4 Hz), 7.04 (2H, d, *J*=8.4 Hz), 6.61 (1H, s), 3.97 (3H, s), 3.88 (3H, s), 3.78 (3H, s), 3.71 (3H, s).

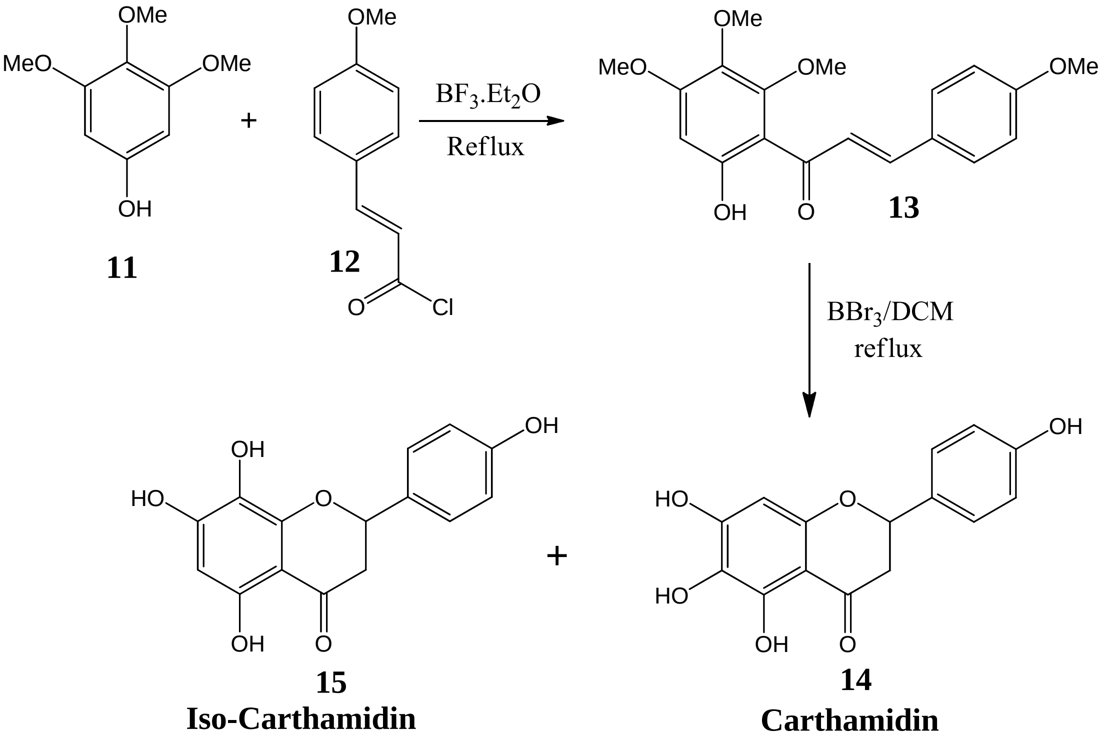

Carthamidin (**14**): To a solution of **13** (2.6 g, 5.2 mmol) in CH_2Cl_2 (40 mL), 1.0 M solution of BBr_3 in CH_2Cl_2 (40.0 mL) was slowly added, then refluxed for 2 h. After cooling to room temperature the reaction mixture was quenched with water and extracted with CH_2Cl_2 then 5% MeOH and EtOAc. Both organic layers were dried on $MgSO_4$ and evaporated under reduced pressure. The resulting pale yellow precipitate was recrystallized from MeOH, yielding 1.01 g pale yellow solid (46%) which was further purified by HPLC to give Carthamidin **(14)** and Isocarthamidin **(15)**. ^{1}H-NMR (CD_3-OD) Carthamidin **(14):** δ 7.31 (2H, d, *J*=6.0 Hz), 6.82 (2H, d, *J*=8.8 Hz), 5.97 (1H, s), 5.30-5.26 (1H, dd, *J*=2.8, 13.6 Hz), 3.12-3.04 (1H, dd, *J*=12.8, 16.8 Hz), 2.7-2.65 (1H, dd, *J*=2.8, 16.8 Hz). ^{1}H-NMR (CD_3-OD) Isocarthamidin **(15):** δ 7.37 (2H, d, *J*=6.4 Hz), 6.82 (2H, d, *J*=6.8 Hz), 5.94 (1H, s), 5.40-5.36 (1H, dd, *J*=2.8, 12.0 Hz), 3.18-3.10 (1H, dd, *J*=12.4, 17.2Hz), 2.75-2.70 (1H, dd, *J*=3.2, 17.2 Hz).

References to Supplemental Experimental Procedures

1. Xia, H., Qiu, F., Zhu, S., Zhang, T., Qu, G., Yao, X. (2007) Biol Pharm Bull **30**, 1308-16
2. Woo, E. R. and Piao, M. S. (2004) Arch Pharm Res. **27,** 173-176
3. Jay, M. and Gonnet, J-F. (1973) Phytochemistry **12**, 953-954
4. Obara, H., Onodera, J., Kurihara, Y., Yamamoto, F. (1978) Bull Chem Society of Japan **51**, 3627-3630
